# Flashbacks Through Time: Historical Development and Current Use of the Term “Flashback” in Psychedelic and Post‐Traumatic Stress Disorder Research

**DOI:** 10.1176/appi.prcp.20250129

**Published:** 2026-03-03

**Authors:** Ricarda Evens

**Affiliations:** ^1^ Department of Psychology Humboldt‐Universität zu Berlin Berlin Germany

## Abstract

In psychiatry, the term flashback is widely recognized in relation to post‐traumatic stress disorder (PTSD) as well as in the characterization of post‐psychedelic experiences. Although it is now most commonly associated with PTSD, the term was originally introduced in the context of psychedelics. While it broadly denotes specific forms of re‐experiencing in both fields, its precise meaning differs significantly between them. In PTSD, flashbacks refer to intrusive, vivid memories with a pronounced “here‐and‐now” quality. In psychedelic research, however, the term flashback does not function as a clearly defined symptom but rather as an umbrella term encompassing multiple similar yet distinct forms of post‐acute psychedelic experiences that potentially arise from different causal mechanisms. This terminological ambiguity complicates systematic investigation and precise clinical understanding of post‐psychedelic symptoms, ultimately limiting effective care for affected individuals. As research into the classification and treatment of post‐psychedelic difficulties advances, the development of a more differentiated and clearly defined vocabulary is needed. Greater conceptual clarity would facilitate improved scientific communication, more targeted research, and better clinical care for individuals navigating the often complex aftermath of psychedelic experiences.

In psychiatry, the term *flashback* is widely recognized in post‐traumatic stress disorder (PTSD) as well as in the characterization of post‐psychedelic experiences. While the term broadly denotes specific forms of re‐experiencing in both fields, its precise usage differs significantly across contexts. Particularly in psychedelic research, the term flashback does not function as a clearly defined psychopathological symptom but is instead used to label multiple similar yet distinct phenomena, potentially arising from different causal mechanisms. This terminological ambiguity complicates systematic investigation and the precise clinical understanding of post‐psychedelic symptoms, ultimately limiting effective care for affected individuals.

While observations of delayed reactions to psychedelics had been described earlier (1), the first documented use of the term flashback in a psychiatric context dates back to the late 1960s, introduced by Mardi J. Horowitz. According to personal communication, Horowitz first presented the term in his 1970 book *Image Formation and Cognition* (2), parts of which were published earlier (3). In his book, Horowitz described flashbacks as follows:

Images formed during a drug‐induced state may be reexperienced repeatedly after the drug has worn off. The terms flashback, flashing, and throwback are “hippie” slang and refer to the subjective sensation of unbidden returns of visual images first formed during the drug intoxication but later repeated long after the drugs have worn off. A secondary meaning is that the image is a repetition of a perception long past.

(p. 15)

Horowitz emphasized that flashbacks were most commonly visual but could involve all sensory modalities. In this initial description, he distinguished three types of flashbacks: (1) spontaneous perceptual distortions (e.g., halos, blurred vision, shimmering)—closely resembling what is now described as Hallucinogen Persisting Perception Disorder (HPPD); (2) increased susceptibility to spontaneous imagery, characterized by vivid and less suppressible visual experiences; (3) recurrent unbidden images of actual psychedelic experiences, often accompanied by anxiety and negative affect—closely aligned with how the term flashback is used today in the context of PTSD.

In the 1970s, Horowitz expanded his focus to post‐traumatic stress symptoms, observing that intrusive, repetitive thoughts also followed experimental stress (4, 5). Throughout the 1970s and early 1980s, the term flashback gained traction in the emerging field of PTSD research, which grew considerably following the formalization of the PTSD diagnosis in 1980 (6, 7, 8). Since DSM‐III‐R (1987), flashback phenomena have been consistently listed under PTSD Criterion B (intrusion symptoms), a classification that has remained unchanged in all subsequent DSM editions. In the current DSM‐5‐TR, this section is described as follows:

Dissociative reactions (e.g., flashbacks) in which the individual feels or acts as if the traumatic event(s) were recurring. (Such reactions may occur on a continuum, with the most extreme expression being a complete loss of awareness of present surroundings.).

In PTSD research and clinical practice, the term flashback is now used fairly consistently to describe intrusive memories that create a vivid “here‐and‐now” sensation—essentially a temporary reliving of the traumatic experience.

In contrast, the use of the term flashback in the context of psychedelics has followed a different trajectory. In DSM‐III, flashbacks were referenced in relation to hallucinogens as “recurrent hallucinations after the hallucinogen is no longer present in the body,” listed as a complication under *Hallucinogen Hallucinosis*. The DSM‐III‐R introduced the diagnosis *Posthallucinogen Perception Disorder*, though notably without referring to flashbacks. By DSM‐IV, the diagnosis had evolved into *HPPD (Flashbacks)*, explicitly incorporating the term in its title and significantly influenced by the research work of Henry David Abraham (9, 10). HPPD was defined in criterion A as:

The reexperiencing, following cessation of use of a hallucinogen, of one or more of the perceptual symptoms that were experienced while intoxicated with the hallucinogen (e.g., geometric hallucinations, false perceptions of movement in the peripheral visual fields, flashes of color, intensified colors, trails of images of moving objects, positive afterimages, halos around objects, macropsia, and micropsia).

The DSM‐IV acknowledged that “Some individuals who use hallucinogen report ‘flashbacks’ that are not associated with any impairment or distress,” emphasizing that such experiences are not pathological per se. A diagnosis of HPPD on the other hand requires that symptoms cause significant distress or functional impairment. Since the DSM‐5, the term flashback no longer appears in relation to HPPD, although the diagnostic description of HPPD otherwise remained largely the same, with the exception that under diagnostic features it is now acknowledged that visual disturbances may not only be episodic but can also be continuous.

Crucially, this definition of HPPD‐flashbacks differs from PTSD‐related flashbacks in three key ways. First, while HPPD‐flashbacks are linked to prior use of hallucinogenic substances, they are not causally tied to distressing or traumatic experiences, as would be required for PTSD. Second, although visual symptoms in HPPD can occur episodically—similar to flashbacks in PTSD—many individuals experience them as continuous or chronic alterations in visual perception. Third, whereas PTSD‐flashbacks are defined by the involuntary re‐experiencing of specific past memories, HPPD symptoms do not necessarily involve the re‐living of autobiographical memories; instead, they may consist of perceptual phenomena reminiscent of intoxication that are, however, experienced as altered perceptions of the *present* moment rather than as reproductions of past events. For such cases, an analogy to *tinnitus*—a persistent disturbance that can cause significant psychological distress but is neither memory‐ nor psychosis‐related—may be more appropriate than the comparison to PTSD‐flashbacks. Furthermore, while PTSD‐flashbacks are typically described as involving multimodal sensory qualities, HPPD symptoms are often predominantly visual.

The distinction between PTSD‐like and HPPD‐like flashbacks is also reflected in their differing treatment approaches. Whereas first‐line treatment for PTSD is trauma‐focused psychotherapy, research on HPPD has focused primarily on pharmacological interventions, with only limited attempts to explore psychotherapeutic treatment (11).

In contrast to the DSM's focus on visual symptoms, the ICD‐10 makes only a brief reference to flashbacks under residual symptoms after substance use, emphasizing their episodic and short‐lived nature as duplicates of previous substance‐related experiences.

These varying definitions and diagnostic framings indicate that psychedelic flashbacks do not represent a single, unified phenomenon but are better understood as an umbrella term encompassing a broader spectrum of post‐psychedelic experiences. At a minimal level of differentiation, it would be useful to distinguish between stress‐related re‐experiencing (akin to PTSD‐flashbacks) and persistent perceptual disturbances (as in HPPD). However, case descriptions suggest that an even broader spectrum of flashback phenomena exists—spanning cognitive, emotional, somatic, and perceptual dimensions. For example, users of potent psychedelics such as 5‐MeO‐DMT frequently report brief, spontaneous “reactivations” of drug effects that are often physical in nature and not necessarily associated with trauma or distress. On the contrary, these episodes are often described as benign or even subjectively positive—sometimes referred to colloquially as a “free trip” (12). While these phenomena share certain characteristics—namely the experience of sensations similar to or reminiscent of previous psychedelic states—they may differ in their underlying causal mechanisms and, when treatment is needed, may require different therapeutic approaches.

Previous research on psychedelic flashbacks has often failed to clearly define the specific nature of the phenomenon under investigation. This lack of conceptual clarity has likely contributed to the divergent—and at times contradictory—findings in studies examining their treatment. To move the field forward, more precise and consistently applied definitions are needed to enable clearer investigation of underlying mechanisms and more targeted therapeutic strategies.

Differentiating between distinct types of flashbacks will allow clinicians to more effectively diagnose, support, and treat individuals experiencing post‐psychedelic challenges. For example, PTSD‐like psychedelic flashbacks, marked by intrusive, emotionally charged memories, may respond well to trauma‐focused psychotherapies. In contrast, HPPD‐related post‐psychedelic symptoms may benefit from management strategies akin to those used in treating tinnitus.

In summary, while the term flashback is today most commonly associated with PTSD—where it refers to intrusive, vivid memories with a strong “here‐and‐now” quality—it was originally introduced in the context of psychedelic experiences. In that field, it functions as an umbrella term encompassing various forms of post‐acute psychedelic experiencing. As research into the classification and treatment of post‐psychedelic difficulties progresses, the development of a more precise and differentiated vocabulary is needed. Such clarity would facilitate better communication, more targeted research, and improved clinical care for individuals navigating the often complex aftermath of psychedelic experiences.

## References

[1] Cooper HA . Hallucinogenic drugs. Lancet. 1955;265(6873):1078–1079. 10.1016/s0140-6736(55)91156-9 14368965

[2] Horowitz MJ . Image formation and cognition. East Norwalk: Appleton‐Century‐Crofts; 1970. p. xiii. 351 S. (Image formation and cognition).

[3] Horowitz MJ . Flashbacks: recurrent intrusive images after the use of LSD. Am J Psychiatry. 1969;126(4):565–569. 10.1176/ajp.126.4.565 5806803

[4] Horowitz MJ . Psychic trauma: return of images after a stress film. Arch Gen Psychiatry. 1969;20(5):552–559. 10.1001/archpsyc.1969.01740170056008 5777306

[5] Horowitz MJ . Intrusive and repetitive thoughts after experimental stress: a summary. Arch Gen Psychiatry. 1975;32(11):1457–1463. 10.1001/archpsyc.1975.01760290125015 1200767

[6] Burgess AW , Holmstrom LL . Rape: sexual disruption and recovery. Am J Orthopsychiatry. 1979;49(4):648–657. 10.1111/j.1939-0025.1979.tb02650.x 573970

[7] Shatan CF . The grief of soldiers: Vietnam combat veterans’ self‐help movement. Am J Orthopsychiatry. 1973;43(4):640–653. 10.1111/j.1939-0025.1973.tb00834.x 4577467

[8] Van Putten T , Emory WH . Traumatic neuroses in Vietnam returnees: a forgotten diagnosis? Arch Gen Psychiatry. 1973;29(5):695–698. 10.1001/archpsyc.1973.04200050100017 4773495

[9] Abraham HD . A chronic impairment of colour vision in users of LSD. Br J Psychiatry. 1982;140(5):518–520. 10.1192/bjp.140.5.518 6980680

[10] Abraham HD . Visual phenomenology of the LSD flashback. Arch Gen Psychiatry. 1983;40(8):884–889. 10.1001/archpsyc.1983.01790070074009 6135405

[11] Doyle MA , Ling S , Lui LMW , Fragnelli P , Teopiz KM , Ho R , et al. Hallucinogen persisting perceptual disorder: a scoping review covering frequency, risk factors, prevention, and treatment. Expert Opin Drug Saf. 2022;21(6):733–743. 10.1080/14740338.2022.2063273 35426769

[12] Ortiz Bernal AM , Raison CL , Lancelotta RL , Davis AK . Reactivations after 5‐methoxy‐N,N‐dimethyltryptamine use in naturalistic settings: an initial exploratory analysis of the phenomenon’s predictors and its emotional valence. Front Psychiatry. 2022;13:1049643. 10.3389/fpsyt.2022.1049643 36523876 PMC9745201

